# Cystic lymphangioma of the pancreatic head treated by enucleation: Case report and literature review^☆^

**DOI:** 10.1016/j.ijscr.2022.107715

**Published:** 2022-09-28

**Authors:** Miguel Almeida, Tiago F. Rama, Rui Quintanilha, Joana Mendes, Vitor Carneiro

**Affiliations:** a General Surgery Department, Hospital do Divino Espírito Santo de Ponta Delgada, Portugal; b Pathology Department, Hospital do Divino Espírito de Ponta Delgada, Portugal

**Keywords:** Incidentaloma, Cystic lymphangioma, Pancreas, D2-40, Local excision, EUS

## Abstract

**Introduction:**

The widespread use of imaging methods has led to an increased identification of asymptomatic Pancreatic Cystic Lymphangiomas (PCL), a rare entity for which available information is very limited.

**Presentation of case:**

We present the case of an asymptomatic 61-year-old male, submitted to elective enucleation of a pancreatic head PCL at our institution. After four years of follow-up the patient is doing well and has no clinical or imaging signs of recurrence.

**Discussion:**

Though rare, PCL should be included in the differential diagnosis of pancreatic cystic neoplasms. All efforts should be made to ascertain a preoperative diagnosis, as expectant follow-up could be a reasonable approach in asymptomatic patients and/or poor surgical candidates. In the face of an uncertain diagnosis, complete surgical excision may be the treatment of choice.

**Conclusion:**

The medical community worldwide should be encouraged to report all cases of PCL, as to increment the overall knowledge about this lesion.

## Highlights

• Widespread use of cross-sectional imaging increased identification of asymptomatic pancreatic cystic lesions.
• Pancreatic Cystic Lymphangioma is one of those rare cystic neoplasms for which available information is very limited.
• Definitive diagnosis often requires pathologic examination of surgical specimen, immunohistochemically with positivity for D2-40.
• En bloc resection with negative margins is curative, however, treatment for asymptomatic cystic lymphangiomas is still controversial.

## 1 Introduction

Widespread use of cross-sectional abdominal imaging has resulted in increased identification of pancreatic cystic lesions in asymptomatic patients [1], [2]. Pancreatic Cystic Lymphangioma (PCL) is one of those rare cystic neoplasms for which available information is very limited.

Lymphangiomas are a benign form of lymphatic malformations that lead to blockage of local lymph flow and lymphangiectasia [2], [3], [4], [5]. A well-established theory suggests that lymphangiomas arise from sequestrations of lymphatic tissue during embryologic development [1], [3], [5]. Abdominal trauma, inflammation, surgery or radiation therapy may lead to lymphatic obstruction and secondary formation of such a tumour [3], [4], [5]. The vast majority of lymphangiomas can be found in the neck region (75 %) and axillary (20 %) and only a small percentage (<1 %) appears in the mesentery region or in the retroperitoneal space. Lymphangiomas arising from the pancreas are extremely rare [1], [2], [3].

Although these lesions are benign, they can often present a diagnostic dilemma as imaging findings are characteristic and can point to the diagnosis, however, confirmation with fine needle aspiration and histopathologic correlation is necessary [1], [2], [3], [4], [5]. Also, the treatment is extremely various; it consists from en bloc resection to simple observation and more or less tight follow up [1], [3], [4]. This work has been written in accordance with the SCARE criteria [6].

## 2 Presentation of case

**Figs. 1:**
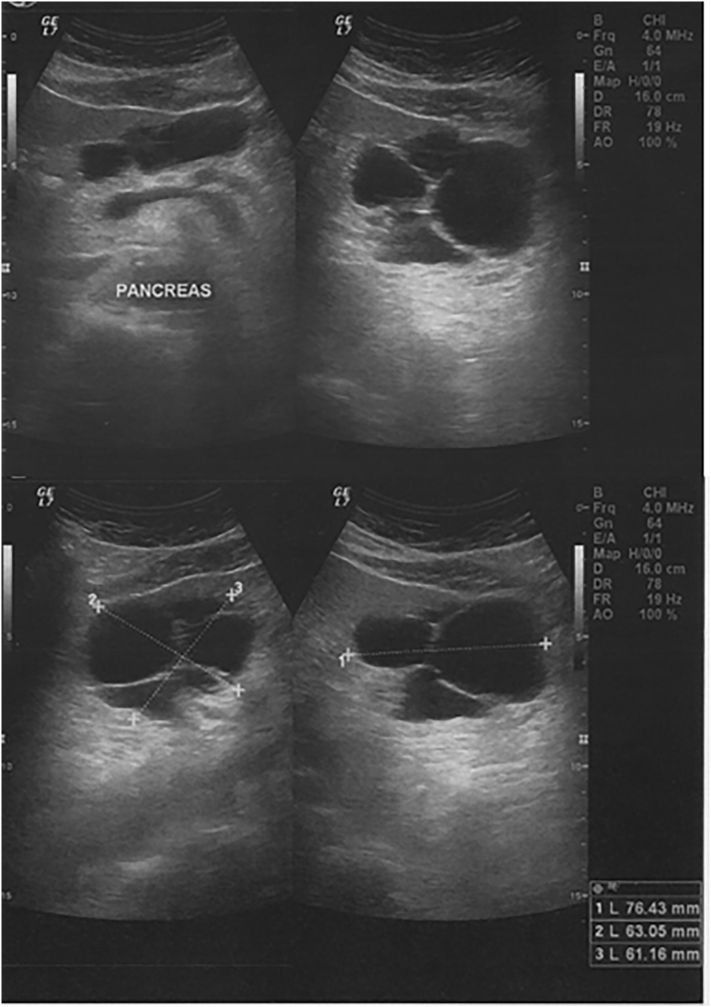
Ultrasound showing a polycystic lesion with fine septa, measuring approximately 76 × 63 × 61 mm, without calcifications or vegetations, located between the pancreas and the left liver lobe.

**Figs. 2:**
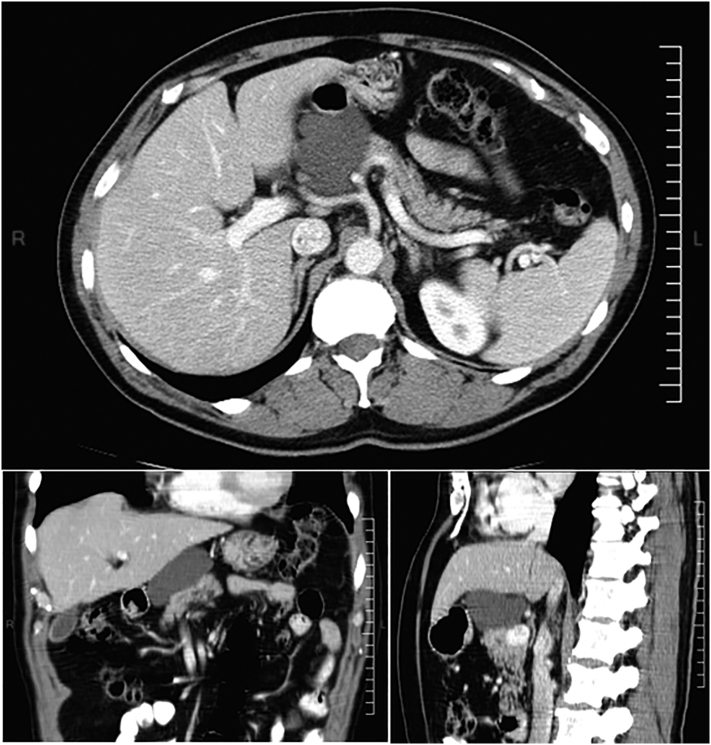
CT abdominal scan (axial, coronal and sagital planes). The nodular cystic lesion located in the head/proximal body of pancreas, with no post contrast enhancement.

A 61-year-old asymptomatic male, without significant past medical history, in particular abdominal trauma or pancreatitis, was referred to our institution after the incidental finding, during a routine renal ultrasound (US), of a polycystic lesion between the pancreas and the left liver lobe, with anechoic content and fine septa, measuring 76x63x61 millimetres (Fig. 1). Computed tomography (CT) scan (Fig. 2) characterized the lesion as being on the dependence of the pancreatic head and proximal body (which was otherwise of unaltered morphology); no local invasion, lymphadenopathy or ascitis was detected. No laboratory abnormalities were found.

Endoscopic ultrasound (EUS) further revealed absence of associated wall thickening, mural nodules, solid masses or connection with main pancreatic duct. A 22-gauge EUS-FNA was performed to aspirate approximately 5 mL of a yellow-white thick fluid, with CEA level of 0,6 ng/mL, Amylase level of 297 U/L; a triglyceride level was unable to be obtained due to partial solidification of material prior to analysis. Cytology was negative for malignancy and showed only scattered mature lymphocytes and no epithelial cells.

**Fig. 3:**
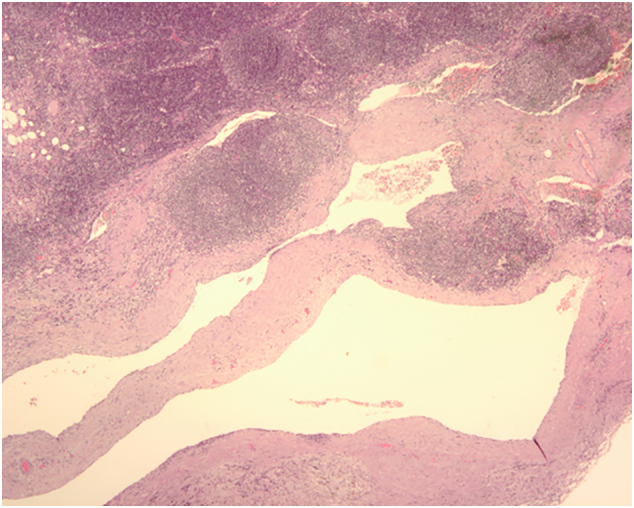
Haematoxylin and eosin staining showed that the multicystic lesion was composed of irregular dilated spaces separated by a stroma of connective tissue (with aggregates of mature lymphoid cells), lined by a layer of endothelial cells, (20×, H&E stain).

**Fig. 4:**
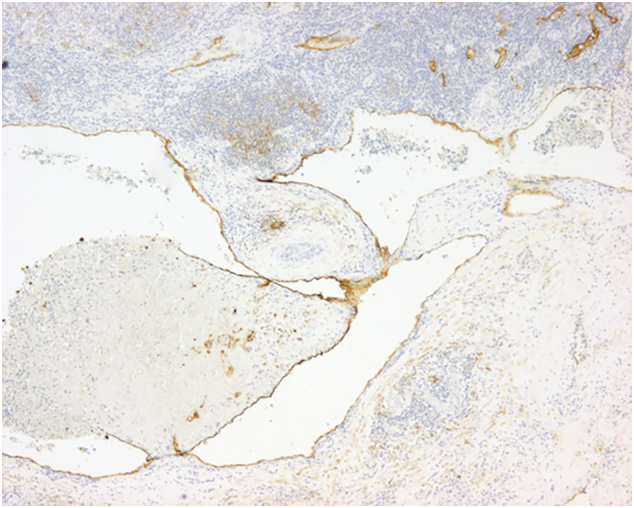
The endothelial cells showed positive staining for D2–40 (20×, D2–40 immunohistochemistry).

Given the volume of the lesion, uncertainty of the diagnosis and patient's preference, he was admitted for surgical exploration, performed by hepatobiliopancreatic surgeon. After a right subcostal incision, with no evidence of locally advanced or metastatic disease, we proceeded to enucleation of the lesion. Fresh frozen sections confirmed benign disease and complete resection. Pathology report a multilocular tumour with 2 to 3 cm cysts filled with yellow-white bloody fluid and, microscopically (Fig. 3, Fig. 4), with walls and septa made of connective tissue with some lymphoid follicles and lymphatic endothelial lining (strongly reactive to D2–40 immunohistochemistry), classifying the tumour as a Pancreatic Cystic Lymphangioma.

Patient had uneventful postoperative course in the surgical ward, with mobilization and oral intake initiated in first postoperative day, discharged at the 7th postoperative day. Four years of follow-up have now been completed with no evidence of recurrence or other complaints.

## 3 Discussion

Pancreatic lymphangiomas comprise <1 % of all lymphangiomas [1], [3], [4], [5], [8], [9], [10] and 0.2 % of all pancreatic neoplasms [4]. Literature is limited mostly to case reports with very few case series [3], adding to a total of 60–100 cases [1], [4], [5], [9]. Female predominance is reported [1], [2], [3], [4], [5], [7], [8], [9], [10], with equal presentation among all age groups [3], [9], [10] and mean age of presentation estimated between 25,3 and 43,1 years [1], [2], [7], [10].

PCL is more commonly located in the body and tail of the pancreas [3], [6], [8], [9], [10], only 17 previously reported cases of this tumour arising from the pancreatic head [3] The size mostly ranges 3–20 cm (average diameter ~ 12 cm) [1], [9], [10].

The clinical presentation is nonspecific, sometimes related to compression of neighbouring organs, often including vague abdominal pain and a palpable mass. Pedicle torsion, rupture, infection or haemorrhage into the lymphangioma may cause acute abdominal symptoms. As in our case, a growing number of patients reported were asymptomatic with incidental diagnosis of lymphangioma on physical examination or radiographic studies for unrelated diseases [1], [2], [3], [4], [8], [9], [10].

The cystic nature of the lymphangioma encompasses a broad differential diagnosis (Table 1).

**Table 1:** Noninclusive list of differential diagnosis of Pancreatic Cystic Lymphangioma.

Type of lesion	Classification	Entity	Reference
Nonneoplasic	*Inflammatory*	Pseudocysts	[1], [2], [3], [4], [5], [9], [10]
		Ductal ectasia in chronic fibrosing pancreatitis	[10]
	*Infectious*	Echinococcal cysts of the pancreas	[4], [8], [10]
	*Inherited*	Polycystic pancreas associated with polycystic kidney disease	[10]
		Von Hippel–Lindau syndrome	[10]
		Cystic fibrosis	[10]
		Renal, pancreatic and hepatic dysplasia sequence	[10]
	*Other*	Lymphoepithelial cyst	[1], [7]
Neoplasic	*Benign*	Serous cystadenomas	[1], [2], [3], [4], [5], [6], [7], [8], [9], [10]
		Mesenchymal tumors (e.g. hemangiomas)	[10]
	*Premalignant and malignant lesions*	Intraductal papillary mucinous neoplasm	[4], [7], [9], [10]
		Mucinous cystic neoplasms	[2], [3], [4], [5], [7], [9], [10]
		Cystic adenocarcinomas	[1], [10]
		*Solid-pseudopapillary tumour*	[2], [8], [10]
		Pancreatic neuroendocrine neoplasms with cystic degeneration	[2], [8], [10]
		Acinar cell carcinoma with cystic degeneration	[8]
		Pancreatic ductal carcinomas with cystic degeneration	[1], [3], [5]

Significant laboratory abnormalities – including serum amylase, CA19.9 and CEA – are absent [1], [2], [3], [4], [5], [7], [8], [9], [10]. Imaging investigations can suggest the diagnosis of cystic lesion and its pancreatic origin [2], as well as relationship with surrounding structures [5], but are often unable to specifically differentiate PCL from all the possible cystic-like tumors of the pancreas [1]. Typical findings and possible advantages of different cross-sectional imaging methods are listed on Table 2.

**Table 2:** Typical findings and possible advantages of the different methods of cross-sectional imaging on Pancreatic Cystic Lymphangioma.

Imaging method	Typical findings and possible advantages	Reference
Abdominal radiograph	Bowel dislocation or obstruction	[3], [5]
US/CT	Well-circumscribed, encapsulated, hypoechoic / water-isodense, uni- or more often multilocular mass with thin septa.	[1], [3], [4], [5], [7], [8], [9]
	Wall and septa may show enhancement by intravenous contrast medium (CT).	[7], [9]
	Rarely, phlebolith-like calcifications can occur in the dilated lymphatic spaces.	[1]
	No ascitis or lymphadenopaty.	[2], [3], [8], [9]
	No evidence of pancreatic or biliary duct dilation.	[2], [7]
MRI	Well-circumscribed, encapsulated, uni- or more often multilocular mass with thin septa, hyperintense on T2-WI and hypointense on T1-WI	[1], [2], [7]
	Superiority to CT in defining interfaces with adjacent structures	[2], [7]
	Superiority to CT in ruling out communication between cyst and pancreatic duct	[1], [2], [7]
	Post‑gadolinium studies have sometimes the advantage of defining the thin septa, when contrast-enhanced CT could not demonstrate.	[2]
18-FDG-PET/CT	Peripheral, low uptake of the radiotracer, with no sign of metabolic activity into the mass	[4]

Preoperative diagnosis using EUS-FNA has been reported, based on: gross appearance of the aspirated fluid (chylous); elevated triglyceride levels (<3000 mg/dL) in the cyst aspirate, with or without the presence of numerous lymphocytes; or the characteristic tissue architecture on paraffin embedded cell-blocks from the aspirates [4], [8], [9]. As in this case, triglyceride level may be unable to obtain due to partial solidification prior to analysis [8]. Analyzing fluid for amylase, CEA and cytology can help further narrow the differential [8].

Definitive diagnosis of PCL often requires pathologic examination of surgical specimen [3], [4], [5], [8], [10]. Gross examination should show a cystic, lobulated mass with variable-sized cystic spaces (that may connect with each other) filled with serous, serosanguineous or chylous fluid. Microscopically, multiple dilated cystic spaces containing eosinophilic, proteinaceous fluid are separated by thin-walled hypocellular septa lined by endothelial cells. Lymphoid aggregates can be present in the lumen and the septa [1], [2], [3], [5], [6], [7], [8], [9], [10]. No cell atypia is found [3]. Surrounding pancreatic tissue may show atrophy and inflammatory changes [5], [10], and no connection with the pancreatic main duct is observed [3], [5]. Dissected lymphnodes should be intact [8]. As in our case, the diagnosis of PCL can be supported immunohistochemically with positivity for D2–40, reliable for identification of lymphatic endothelium [3], [4], [5], [10].

En bloc resection with negative microscopic margins is curative [1], [3], [4], [9], [10] and should be offered to symptomatic patients and those with complications [3], [4], [7], [8], [9]. Laparoscopic approach is feasible in selected cases [7], [9]. PCL can behave in an aggressively locally invasive manner and grow to an enormous size, imposing multi-organ resection [3], [4], [5], [7], [8], [9]. Incomplete excision is associated with recurrent disease (10 %–50 % rate) [1], [3], [4], [7], [8], [9]. Treatment for asymptomatic cystic lymphangiomas is still controversial [1], [3], [7]. Certain cases with no treatment had no significant progression on radiological follow-up [1], [3]. In this setting, further treatment may be unnecessary, making an acceptable approach to follow the patient clinically along with periodic imaging [7]. In our case, an uncertain preoperative diagnosis and patient's preference, in a good surgical candidate prompted us to surgery.

## 4 Conclusion

In this study, we report a very rare case of cystic lymphangioma of the head of the pancreas. Despite being benign and mostly asymptomatic, we believe that it should be considered in the differential diagnosis of cystic tumors of the pancreas. Complete surgical excision has the dual advantage of allowing definitive diagnosis and treatment, however, asymptomatic cases with no suspicion of malignancy in the diagnostic study, observation with clinical and radiologic follow-up may be favoured.

## Sources of funding

Authors did not receive funding for this research.

## Ethical approval

Not applicable.

## Consent

Written informed consent was obtained from the patient for publication of this case report and accompanying images. A copy of the written consent is available for review by the Editor-in-Chief of this journal on request.

## Registration of research studies

Not applicable.

## Guarantor

Miguel Almeida

## Provenance and peer review

Not commissioned, externally peer-reviewed.

## CRediT authorship contribution statement

All authors namely, Dr. Rui Quintanilha, Dr. Vitor Carneiro, Dr.ª Joana Mendes, Dr. Tiago Rama e Dr. Miguel Almeida were involved in the management of this patient. All authors contributed equally to the design of the work and acquisition, analysis and interpretation of data. Dr. Miguel Almeida and Dr. Tiago Rama drafted the manuscript. Dr. Rui Quintanilha, Dr. Vitor Carneiro and Dr.ª Joana Mendes substantively revised it. All authors read and approved the final manuscript.

## Declaration of competing interest

The authors declare that they have no competing interests.
